# Optimizing the Prediction Process: From Statistical Concepts to the Case Study of Soccer

**DOI:** 10.1371/journal.pone.0104647

**Published:** 2014-09-08

**Authors:** Andreas Heuer, Oliver Rubner

**Affiliations:** 1 Institute of Physical Chemistry, WWU Muenster, Muenster, Germany; 2 Center of Nonlinear Science CeNoS, WWU Muenster, Muenster, Germany; University of California Irvine, United States of America

## Abstract

We present a systematic approach for prediction purposes based on panel data, involving information about different interacting subjects and different times (here: two). The corresponding bivariate regression problem can be solved analytically for the final statistical estimation error. Furthermore, this expression is simplified for the special case that the subjects do not change their properties between the last measurement and the prediction period. This statistical framework is applied to the prediction of soccer matches, based on information from the previous and the present season. It is determined how well the outcome of soccer matches can be predicted theoretically. This optimum limit is compared with the actual quality of the prediction, taking the German premier league as an example. As a key step for the actual prediction process one has to identify appropriate observables which reflect the strength of the individual teams as close as possible. A criterion to distinguish different observables is presented. Surprisingly, chances for goals turn out to be much better suited than the goals themselves to characterize the strength of a team. Routes towards further improvement of the prediction are indicated. Finally, two specific applications are discussed.

## Introduction

Panel data analysis deals with a regression procedure where individual subjects as well as information at different times is taken into account [1]. The update of estimators with time can be related to Bayesian approaches [2], [3] as explicitly discussed, e.g., in [4]. For Gaussian statistics there exists a direct connection between Bayesian inference and a regression analysis; see, e.g., [5]. Actually, Bayesian inference to soccer has recently been discussed in Ref. [6].

Of key interest is the knowledge about the quality of the estimator. Here we simplify the general result by using the assumption that the underlying property of the subject does not change between the final measurement and the prognosis time interval. This does not necessarily hold for the time of earlier measurements. However, due to the random noise, by which the most recent measurement may be disturbed, it may still be favorable to take into account older pieces of information. Having an explicit expression of the estimator quality it is possible to judge the relevance of the available information for the prediction process in a detailed manner. Furthermore, we can define the limit of optimum prediction and judge, how far a specific prediction procedure differs from this limit.

We apply this approach to the prediction of soccer matches but we expect that it may have a broader applicability for many different types of sports and beyond where the future achievements of, generally speaking, different subjects is constant between the most previous measurement and the near future.

To set the present approach into perspective, we would like to summarise some specific approaches for soccer prediction. In one type of models [7]–[10] appropriate parameters are introduced to characterise the properties of individual teams such as the offensive strength. Of course, the characterisation of team strengths is not only restricted to soccer; see, e.g., [11]. The specific values of these parameters can be obtained via Monte-Carlo techniques. These models can then be used for prediction purposes and allow one to calculate probabilities for individual match results. A key element of these approaches is the Poissonian nature of scoring goals [12]–[14]. Beyond these goals-based prediction properties also results-based models are used. Here the final result (home win, draw, away win) is predicted from comparison of the difference of the team strength parameters with some fixed values [15]. The quality of both approaches has been compared and no significant differences have been found [16]. Going beyond these approaches additional covariates can be included. For example home and away strengths are considered individually or the geographical distance is taken into account [16]. Recently, also the ELO-based ratings have been used for the purpose of forecasting soccer matches [17]. Recent studies suggest that statistical models are superior to lay and expert predictions but have less predictive power than the bookmaker odds [17]–[20]. This observation strongly suggests that either the information, used by the bookmakers, is more powerful or, alternatively, the inference process, based on the same information, is more efficient. Probably, both aspects may play a role.

The structure of this paper is as follows. First, we introduce the statistical background of prediction. In particular we show that under the general assumptions, mentioned above, the quality of the estimation can be determined in simple analytical terms. Then this general scheme is applied to the prediction of soccer matches, using the German premier league (Bundesliga) as an example. It can be shown that all assumptions, used in the previous Section, are fulfilled to a very good approximation. Furthermore, it is shown that chances for goals possess a very high information content about the individual team strengths and are, thus, chosen for the respective covariates. Subsequently, the theoretical results are compared with the explicit bivariate regression analysis. The specific setting is chosen such that one wants to predict the outcome of the second half of a season, based on knowledge of a variable number of matches from the first half of the same season as well as all matches of the previous season. In particular we discuss the dependence of the prediction quality on the number of matches, taken into account. Furthermore, it is shown, how the present concepts can be applied to the prediction of single matches. We end with a discussion.

## The Statistical Background of Prediction

### Variables

**Figure 1:**
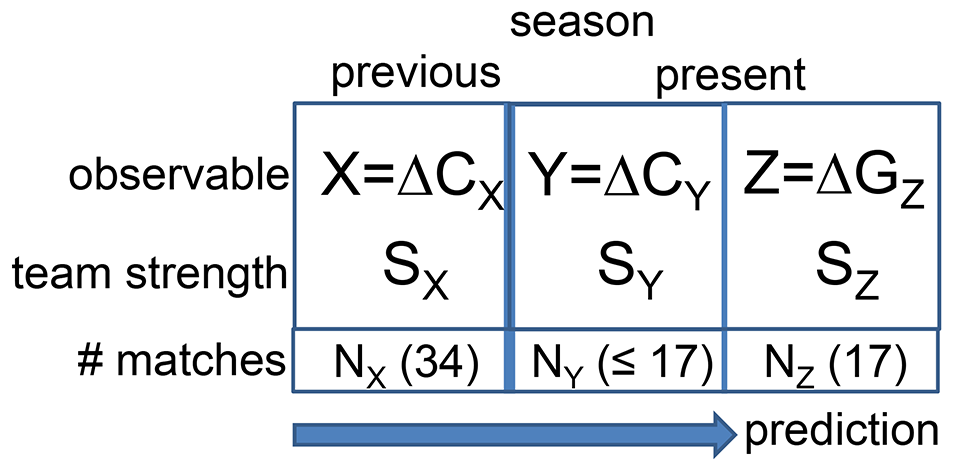
Schematic representation of the general prediction setup.

We consider two successive time intervals, in which we measure the independent variables 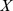 and 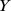. For the later application to soccer this might be the accumulated goal difference during the previous season and during the present season, measured individually for each team. Here we consider differences in order to capture both the offensive and defensive strength. Specifically, we perform this analysis after half of the present season is over. Naturally, this can be easily generalized to other situations. The aim is to predict the goal difference 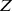, i.e. the dependent variable, of each team during the second half of the season. This setup is sketched in Fig.1. The prediction quality can be explicitly expressed and compared with the theoretical optimum.

### Regression

First, we briefly review some key relations of regression analysis. We start with the linear relation 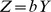 for the independent variable 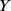 and the dependent variable 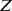. Note that we assume all variables fulfill the condition that their first moment is zero. Generalisation is, of course, straightforward. The regression problem requires the minimisation of 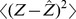 with respect to 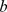 where 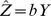 is the predictor of 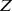. Substituting the resulting value of 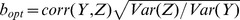 yields for the optimum quadratic variation, denoted 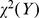,
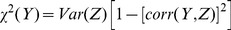
(1)where 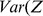) denotes the variance of the distribution of 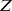 and
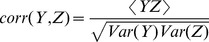
(2)is the Pearson correlation coefficient between the variables 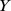 and 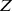. Eq.1 has a simple intuitive interpretation: The higher the correlation between the variables 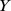 and 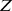, the better the predictability of 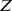 in terms of 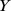.

For the present work we are mainly dealing with the bivariate regression 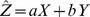. Via normal equations, one can obtain general expressions for the regression coefficients 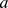 and 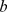. Interestingly, the prediction quality of the bivariate prediction can be analogously expressed to Eq.1 and reads
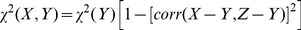
(3)where the partial correlation coefficient
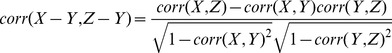
(4)is used and 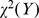 is defined as in Eq.1. The second factor on the right-hand side of Eq.3 explicitly contains the additional information of the variable 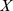 as compared to 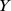. One can easily show that in agreement with expectation Eq.3 is completely symmetric in 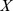 and 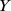.

Here we present a straightforward derivation of Eq.3. Let 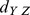 denote the solution of the regression problem 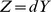. Accordingly, 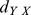 is the solution of the regression problem 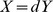. In a next step one defines the new variables 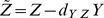 and 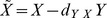. For these new variables the correlation with 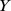 is explicitly taken out. A straightforward calculation shows that the Pearson correlation coefficient 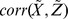 is exactly given by the partial correlation coefficient 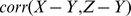.

Now we consider the regression problem of interest 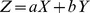. In a first step it is formally rewritten as
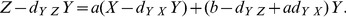
(5)Using the above notation and introducing the new regression parameter 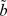 we abbreviate this relation via
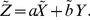
(6)By construction the observable 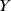 is uncorrelated to 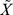 and 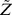. Therefore the independent variable 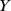 does not play any role for the prediction of 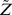 so that effectively one just has a single-variable regression problem. Therefore one can immediately write
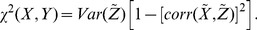
(7)The first factor is identical to 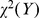 whereas the Pearson correlation coefficient in the second factor is identical to 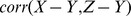. This concludes the derivation of Eq.3.

### Prediction for individual subjects/teams

As introduced, the variables 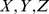 denote the output of a team or, more generally, of some subject during three successive time intervals. For the first time interval, the outcome of team 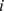 is denoted 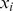. Conceptually, this value has contributions from the true underlying team strength 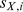 as well as from random non-predictable effects 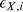, i.e.
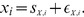
(8)Thus, only in the absence of random effects the team strength 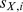 could be directly identified with the outcome 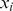. In what follows we use the terminology of soccer but this approach can be directly applied to other cases where the observable is the sum of the properties of the respective subject and some random effects.

Following the previous discussion we only consider observables 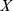 for which the first moment disappears after averaging over all teams. Naturally, the same holds for the team strength observable 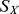. Squaring Eq.8 and averaging over all teams yields
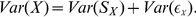
(9)Analogous relations hold for 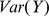 and 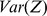.

For the evaluation of the prediction quality Eq.3 one needs to calculate individual correlations such as 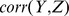. A straightforward calculation yields
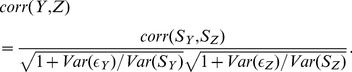
(10)Again, analogous expressions hold for 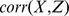 and 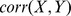.

Eq.10 allows one to identify two distinct reasons why the correlation of 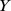 and 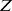 is smaller than unity. First, the team strength may change between the two time intervals, i.e. 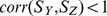. Second, the random effects, which influence the observables 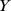 and 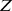, may play an important role (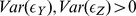).

The subsequent discussion is based on the mathematical identity
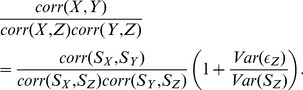
(11)As a first step of simplification we want to estimate the team strength 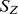 rather than 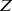 itself. Then the prediction quality is denoted by 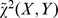. All relations remain identical except 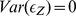 in the evaluation of quantities, occurring in Eq.3. Naturally, one has the simple relation
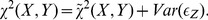
(12)As the second step we consider the special case that the team strengths are the same in the second and third time interval, belonging to 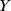 and 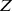, respectively. Actually, it has been already shown in Ref. [21] that apart from short-time fluctuations the team strength remains constant during the course of a season. As a consequence one has 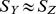, i.e. nearly the same team strength in the first and the second half of a season. Mathematically, we assume a strict equality. The corresponding empirical result will be discussed further below. A mathematical consequence is (see below for specific data) 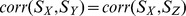. Under this assumption, Eq.11 can be rewritten as
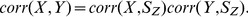
(13)


Inserting this relation into Eq.3 for the prediction quality of 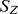 the general expression simplifies significantly and one obtains
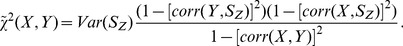
(14)


This is the key relation to be used when estimating the quality of the prediction. Apart from the assumption of constant properties during the final two time intervals, this relation is generally valid.

## Application to the Case of Soccer Prediction: Concepts

### General

Our general goal is the prediction of the future results of soccer matches. Specific data are taken for the German premier league (Bundesliga), employing information about all matches between the seasons 1995/96 and 2010/11. During a season a team has 34 matches.

Our goal is the prediction of the aggregated results 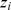 of each team 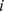 of the second half of a season, based on knowledge about 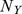 match results 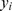 from the first half of the season as well as the 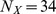 results 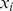 from the previous season. As the dependent variable 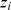 we choose the goal difference but a similar analysis could be also performed for points; see again Fig.1. Of course, due to the generality of our approach also different prediction problems can be handled. For the explicit calculations of the goal differences we correct for the home advantages [5] so that the statistical properties are independent of the home advantage.

### Disentangling random and systematic effects

For our analysis it is essential to decompose the variables 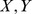 and 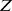 into its systematic parts (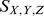) and its random contributions (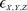); see Eq.8. As mentioned above, 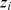 will be identified as the goal difference of team 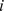 after 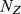 matches, normalised by 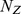. In case of matches under identical conditions the random effects are averaged out as reflected by the standard scaling relation 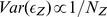 where the proportionality constant is denoted 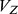. Thus, we have
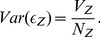
(15)By studying the dependence of 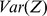 on 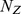 the systematic and random contributions to 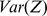, as expressed in Eq.9, can be identified. Of course, analogous relations hold for 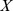 and 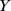.

Strictly speaking, the scaling with the inverse number of the matches breaks down for 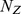 close to unity because then different strenghts of the opponents no longer average out. In practice it turns out that for 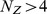 the difference of the 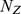 opponents has sufficiently averaged out. This dependence on the number of considered matches has been explicitly analysed in Ref. [5], [22]. For the present set of data we obtain 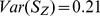 and 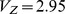. Actually, 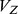 is very close to the total number of goals per match (2.85). This expectation is compatible with the assumption of independent Poisson processes.

### Choice of observables

The goal is to predict the goal difference 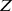 or, alternatively, the team strength 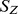. A natural choice for the independent variables 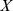 and 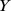 are the goal differences in the respective time intervals. In what follows, goal differences are denoted as 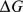. However, as will be shown below, this choice is far from optimum. Generally speaking, one aims for observables which contain as much information as possible about the team strength.

How to capture the information content of a given observable? For this discussion we restrict ourselves to the prediction problem 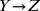 to be solved via a simple univariate regression as summarised above (see Eq.1). For this analysis we use 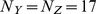, i.e. all matches from the first and second half of the season, respectively. The quality of the prediction is captured by 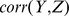. The larger the value 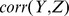, the better the prediction and thus the higher the information content of 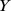 about the team strength. From the empirical data we obtain 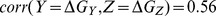.

Can one increase 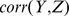 significantly beyond the value of 0.56 by using other observables? The scoring of goals is the final step in a series of match events. One may thus hope that there exist other match characteristics which are even more informative about the team strength. A possible candidate is the number of chances for goals. They are provided by a professional sports journal (www.kicker.de) for all seasons, considered in this work. We denote the chances for goals as 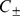 and the goals as 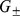. The sign indicates whether it refers to the considered team (+) or the opponent of that team (−).

Next we define the scoring efficiencies 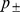 via the relation
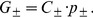
(16)Here, 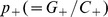 denotes the probability that the team is able to convert a chance for a goal into a real goal and 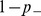 that the team manages to not concede a goal after a chance for a goal of the opponent. Averaging over all teams and seasons one obtains 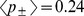. Thus, every forth chance for a goal ends up in a goal.

**Figure 2:**
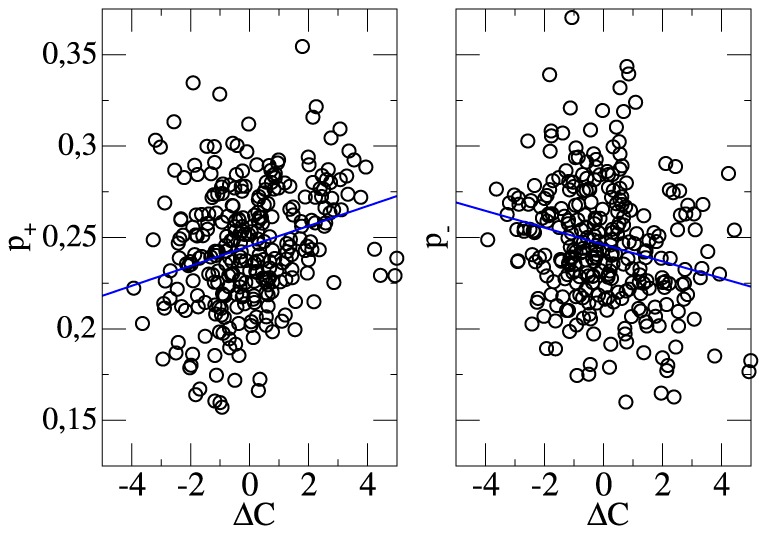
The efficiency factors 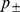 as a function of the differences of the chances for goals 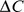.

In Fig.2 the actual scoring efficiencies 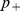 after a season are shown together with the respective values of 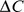. Very clearly, the goal efficiencies are widely distributed between approx. 15% and 35%. On average, better teams with a larger value of 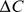 have a slightly better efficiency to score goals and more likely avoid to concede goals (correlation coefficients 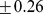). Despite this small correlation, the large scatter of 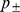 cannot be explained in terms of 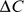.

This large unexplained variance seems to imply that the scoring efficiencies strongly vary from team to team in an a priori unknown way. As a consequence the chances for goals would hardly contain additional information about the expected number of goals, which a team is going to score in the future. In particular, the estimation of the team strength, which is defined on the basis of goals, would hardly be improved by taking into account the chances for goals.

With the definition 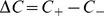 this statement is equivalent to the presence of a weak correlation between 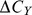 and 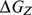. However, this preliminary conclusion is wrong. Rather the correlation coefficient turns out to be 
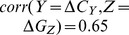
 which is much larger than the value of 
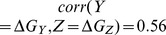
. Stated differently, the chances for goals are by far more informative for the prediction of the team strength than the goals themselves!

### Why chances for goals are so informative

This observation could be rationalized under the hypothesis that the scoring efficiencies are very similar for all teams. Qualitatively, one can argue in this limit that random effects are stronger for goals than for chances for goals, since the number of goals is typically smaller than the number of chances for goals. To quantify this aspect, we consider a simple example of a fictive coin-tossing tournament where the head appears with probability 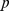 which in this simple example is given by 1/2. A team is allowed to toss the coin 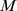 times per round. In the first round this results in 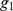 times tossing the head. Thus, in the first round one has observed the number of tosses 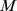 as well as the number of heads 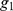. In the relation to soccer 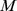 would correspond to the number of chances for goals and 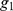 to the number of goals in that match. In order to keep the argument simple we assume that 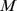 is a constant whereas in a real soccer match 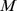 can vary. How to predict the expected number of heads 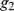 in the next round? Here we consider two different approaches. (1) The prediction is based on the achievement of the first round, i.e. on the value of 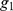. Then the best prediction is 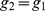. The variance of the statistical error of the prediction can be simply written as 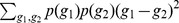 where 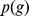 is the binomial distribution. A straightforward calculation yields for this variance a value of 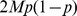. (2) The prediction is based on the knowledge of tossing attempts 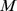. If furthermore the value of 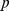 is known the optimum prediction is, of course, 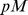. The variance of the statistical error is given by the binomial distribution, i.e. by 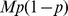. Stated differently, knowing the number of attempts to reach a specific goal (here tossing a head) is more informative than the actual number of successful outcomes as long as the probability 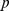 is well known. Note that in this limit the common value of the scoring efficiency is very well determined because it results from averaging over all teams.

**Figure 3:**
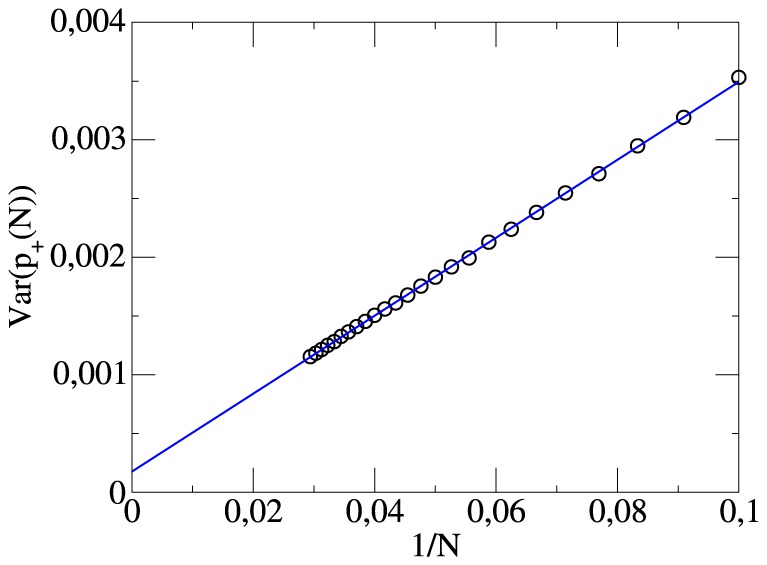
The variance of the distribution of scoring efficiencies in dependence of the number of match days.

This hypothesis seems to contradict the results Fig.2, as presented above. However, a priori the large fluctuations of 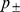 in Fig.2 do *not* necessarily contradict the presence of a rather uniform value of 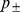 for all teams. Rather, this apparent disagreement can be easily resolved by discussing in more detail the possible reasons for the strong fluctuations of 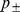 when comparing different teams. In general, these fluctuations are a superposition of two effects: (i) true differences between teams and (ii) statistical fluctuations, reflecting the random effects in the 34 soccer matches of the season. In analogy to the previous discussion both effects can be disentangled by studying the dependence of the variance of 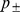 on the number of matches 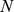, which has been used for the averaging. The results of this analysis is shown in Fig. 3. One can see that the extrapolation to large 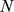, i.e. the systematic team-specific variance of 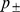, yields a value (0.0002) which is much smaller than the variance for 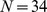 (0.0012), i.e. after averaging over a whole season. Thus, the large fluctuations in Fig.2 are mainly of statistical nature and the efficiency to score a goal from a chance for a goal is basically the same for all teams! We note in passing that to a large extend the residual variance of 0.0002 can be explained via the above-mentioned effects that better teams have a slightly higher scoring efficiency.

Based on this intriguing result we will identify 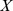 and 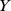 as the differences of the chances of goals in the respective time intervals, denoted 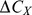 and 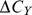. In analogy to Eq.9 and Eq.15 we can identify the disentanglement into systematic and random contributions. The results are listed in Tab.1.

**Table 1:** The different systematic and random contributions of the observables, relevant for this work.

	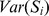	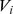
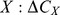	2.32	14.1
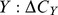	2.66	14.2
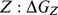	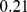	2.95

As expected the statistical characterisation of the random components of 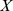 (complete season) and 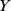 (first half of a season) are very similar because both deal with chances for goals. The small remaining differences express the fact that the statistical properties of the first and the second half of the season are slightly different [22]. Finally, we note in passing (data not shown) that knowledge of the goal differences of 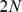 matches has the same information content as knowing the chances of goals of just (approx.) 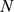 matches.

### General statements about the degree of predictability

In the explicit form of 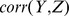 (see Eq.10) all terms on the left and right side except for 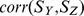 have been quantified so far, either via the information in Tab.1 or via explicit determination of 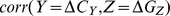, yielding 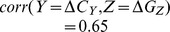 (with the choice 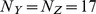). This allows one to determine the correlation between the team strength in the first half and the second half of the league. We obtain 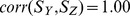. This has two important implications. First, the variation of the team strength during a single season is basically absent, as already reported in [5]. Second, the team strength as defined via the chances for goals (corresponding to 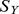) is, apart from a proportionality factor, basically identical to the definition of the team strength as defined via the goals (corresponding to 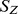). Both results are very promising with respect to the ability to predict soccer matches. In particular, the key approximation, entering Eq.14, is indeed very well fulfilled.

We mention in passing [22] that a closer analysis reveals that the team strength fluctuates with a small amplitude of approx. 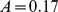 and with a decorrelation time of approx. 7 matches. Since we average over a larger number of matches and, furthermore, restrict ourselves to the prediction of the total second half, these temporal fluctuation are to a large extent averaged out and do not show up in the present statistical analysis.

In case that the team strength 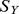 is perfectly known, i.e. 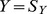, Eq.10 yields (using 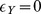) 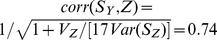. One may compare this limit of optimum prediction with the case where 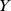 was calculated based on the the chances for goals (correlation of 0.65) or based on the goals (correlation of 0.56). This clearly reveals that using the chances for goals instead of the goals yields a significant step towards the theoretical optimum.

The final unknown in our prediction scheme are values of 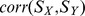 and 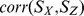 which can be determined in analogy to 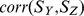. Explicit calculation yields 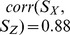 and 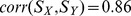. Both values are identical within statistical errors (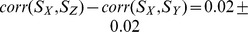). This is compatible with the observation that the team strength does not vary within a season but vary within the summer break. For future purposes we use the average value of 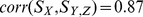 for the characterization of the correlation of the team strength between two seasons.

## Application to the Case of Soccer Prediction: Results

### Prediction of team strength

To check our analytical results we perform an explicit multivariate regression analysis to estimate 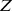 based on knowledge of 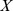 and 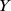 by using standard algorithms. To capture the dependence on the information content of the first half of the present season we also vary the number of considered matches 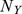. To improve the statistical quality of the data for 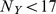 we always average over different random selections of 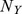 matches from the first half of the season. For the determination of 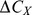 we choose all matches, i.e. 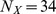 (thus taking the whole season). To check the relevance of the information from the previous season we alternatively set 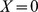, i.e. ignore the information from the previous season.

One technical aspect needs to be mentioned. In a given season two or three teams have just been promoted. Thus, no data about the previous season are available. Therefore, we set the value of 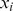 for the differences of the chances for goals for the promoted team to a constant value 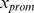. This value is determined by the condition that the resulting average value 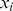 (averaged over all teams of the present season) is zero.

**Figure 4:**
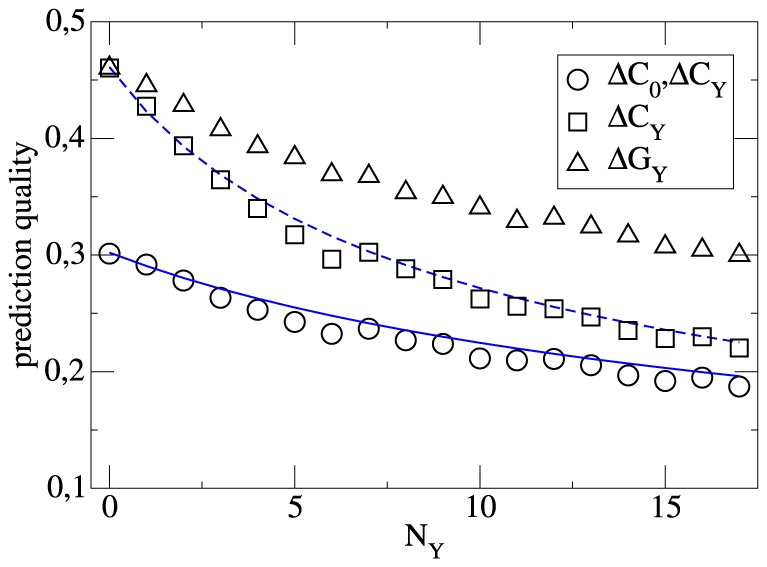
The prediction quality of the team strength, determined via 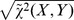, as a function of the number of match days 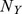. Different choices of variables are shown. For the second and third case 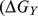 and 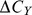, respectively) the information from the previous season is neglected. The solid lines are based on the explicit formulas for the prediction quality.

The numerical results are shown in Fig.4. We start with the case 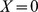. One can see that (trivially) for 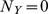 the standard deviation in the estimation of the team strength is identical to the standard deviation of the 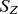-distribution because no team-specific information has been used. The longer the season, the more information is available to distinguish between stronger and weaker teams. Using the information of the complete first half of the season (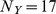) the statistical uncertainty decreases to 0.22.

We have repeated the same calculation by identifying 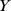 with the goal differences 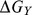. The prediction quality is significantly worse and one obtains an uncertainty of 0.30 rather than 0.22 after 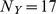 matches.

When additionally incorporating the information from 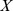, the statistical uncertainty is already quite small at the beginning of the season (0.3). Of course, when increasing 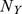 it further decreases. Even after 17 matches the additional gain of using 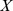 is significant (0.19 vs. 0.22). Thus, despite the slight decorrelation of the team strength during the summer break it is advantageous to take into account the information from the previous season even after half of the present season has been played.

Furthermore, we compare in Fig.4 the actual uncertainty of the prediction of 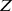 with the theoretical expectation as expressed by Eq.12 and Eq.14. One finds a very close agreement with the actual data. This serves as a consistency check of our whole procedure and just reflects the fact that the assumptions, underlying the derivation of Eq.14, are fulfilled very well.

Finally, we explicitly apply this formalism to the prediction of a specific season of the Bundesliga. We aim to predict the goal difference of the 2nd half based on previous information. The regression problem reads 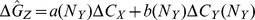 where the weighting factors depend on the number of matches, included from the first half of the present season. They are listed in Tab.2 for different values of 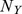. Naturally, for 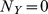 the estimation is only based on 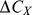. Here the regression coefficient can be also calculated analytically using the values, mentioned in this work. Specifically, one gets 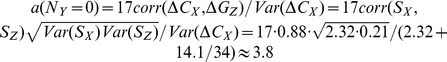 which is very close to the numerically determined value of 3.71. As expected, more information during the present season, i.e. larger 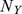, leads to a stronger weighting of 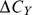. After 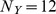 matches the information contents of the previous season is basically equal to that of the first matches of the present season.

**Table 2:** The two regression parameters as a function of 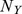.

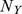	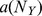	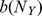
0	3.71	0
4	3.20	0.82
8	2.60	1.70
12	2.23	2.30
17	1.86	2.77

Based on these regression parameters we explicitly predict the goal difference of the second half for the two cases 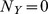 and 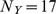. We present data for the season 2007/08. Both predictions for 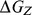 are listed in Tab.3 together with the actual values of 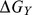 and 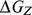 during that season.

**Table 3:** The predictions of the goal difference of the second half of the Bundesliga-season 2007/08 for each team, based on the differences of chances for goals 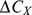 of the previous season (3rd column) or, additionally, on the differences of chances for goals 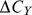 of the first 17 matches of the present season (4th column).

	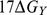	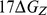	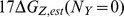	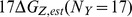	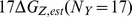
					plus market value
B. München	23	24	10	21	23
Bremen	18	12	11	15	14
Hamburg	11	10	3	9	10
Leverkusen	16	1	2	9	8
Schalke	9	14	8	12	11
Karlsruhe	−2	−13	−8	−6	−7
Hannover	−1	−1	3	−1	−2
Stuttgart	−1	1	9	5	6
Frankfurt	−4	−3	2	−3	−4
Dortmund	−4	−8	0	0	2
Wolfsburg	0	12	−4	−5	−2
Hertha	−5	0	−5	−8	−5
Bochum	−2	−4	−1	−4	−7
Bielefeld	−19	−6	−6	−11	−10
Rostock	−10	−12	−8	−11	−13
Nürnberg	−7	−9	1	1	1
Cottbus	−10	−11	−8	−10	−12
Duisburg	−12	−7	−8	−13	−12

The estimation in the final column also involves information about the market value. The actual goal differences of the first half of that season and the second half are included in the first two columns, respectively.

One can see that for most cases the prediction before the season and in the middle of the season agree quite well, i.e. no dramatic reevaluations of the team strength as compared to the previous year was necessary. Notable exceptions are München (estimation of +10 before the season and +21 after half of the season) and Leverkusen (increase from +2 to +9). Obviously, this reevaluation reflects that the fact that both teams played much better during the first half of that season (goal differences of +23 and +16 for München and Leverkusen, respectively) than expected beforehand.

The final column also contains information about the logarithm of the market value (taken from www.transfermarkt.de) as an independent variable for a trivariate regression problem. The scaling of the team strength with the logarithm of the market value has been explicitly shown in previous work [22]. The resulting modifications in the estimation of 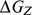 are small but significant. When averaging over all years between 2001/02 and 2010/11, for which the market value is available, it turns out that the prediction quality improves by 0.02 for 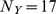. Thus, relative to 0.19 a further significant improvement can be achieved.

### Prediction of single matches

Please note that the estimation of 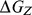 is the basis for many other types of prediction. Since 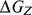 is nothing else than the team strength, this value can be directly taken to estimate individual matches. For example, on the 18th match day of the season 2007/08 Cottbus was playing vs. Leverkusen. As shown in Refs. [21], [22] the expected goal difference during a match of team 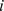 and 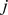 in the Bundesliga is given by the difference of the team strength of both teams plus some team-independent contribution, reflecting the home advantage. Nonlinear effects can be neglected. For this specific match the expected outcome was (using the final column in Tab.3): 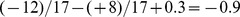, using the home advantage of approx. 0.3 during that season. Thus, the best estimation for the resulting goal difference of that match, based on the available information used in this work, is -0.9. Actually, the final result was 2∶3.

Here is a brief summary of the different prediction steps, following the general procedure in [21] and in agreement with previous work (e.g.[10]).

1. Calculation of the team strength via a linear regression approach. As main parameters enter 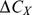, 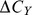, and the logarithm of the market value of the team at the beginning of the season. Naturally, for a match on the M-th match day one uses 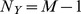. Minor further improvements can be reached by introducing an index for promoted teams and by taking into account short-time fluctuations of the team strength by using the results of the last seven matches as an individual parameter [22]. In total, this ends up in a five-dimensional regression analysis. The regression parameter have been obtained from comparison of all seasons between 1995/96 and 2010/11, excluding the season which predictions are performed.

2. Calculation of the sum of goals by a corresponding regression analysis, taking into account the goals, scored in the present season so far, and the goals of the previous season [21]. However, for the calculation of the outcome of individual matches this step is by far less important than the estimation of the team strength.

3. Estimation of the team-independent home advantage in the corresponding season in analogy to the previous step [22].

4. Calculation of the expectation value of goals of both teams from steps 1–3.

5. Estimating possible final scores by assuming independent Poisson processes.

6. Correcting for the effect that draws are more likely than expected on the expense of matches with goal differences 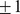 [23].

Note that in earlier work goals rather than chances for goals were employed. We would like to stress again that the critical part of this endeavor is the determination of the team strength as described in this work.

To characterize the quality of the present approach we have compared the predictions of single matches with odds from Oddset, using data between the seasons 2002/03 and 2006/07, where the odds were available to us. Specifically, we used the scaled inverse odds as an estimate of the respective probabilities for a win of the home team, a draw, or a win for the away team. An objective measure is the parameter
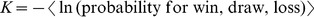
(17)where the probability for the actual outcome is taken as the argument of the logarithm. One can show that the value of 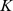 is a minimum if the predicted probabilities for a win, a draw, and a loss are identical to the true probabilities. Analogous measures can be already found in literature, e.g. [10], [24]. One can see in Tab.4 the additional consideration of new information indeed gives rise to a lower value of 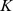. Furthermore, restricting the choice of matches to those taking place during the first 10 match days, the prediction becomes worse (larger 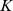). In particular, the additional impact of the market value is larger, if restricting oneself to the first 10 matches of the season. When averaging over all matches in these seasons [22], it turns out that the K-value of the present approach is smaller than the K-value for the Oddset-odds by 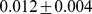. Thus, the comparison yields a highly significant improvement of the present model as compared to the Oddset-odds. The size of this improvement is non-negligible if compared to the variations of 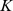 when adding different pieces of information; see Tab.4.

**Table 4:** The K-value for the regression model during the seasons 2002/03 and 2006/07 as well as for the Oddset-odds.

	first 10 matches of season	all 34 matches
Only home advantage	1.073	1.057
+ matches of present season	1.054	1.013
+ matches of previous season	1.027	1.004
+ market value	1.019	1.000
Oddset	1.025	1.012
Difference	0.006 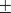 0.009	0.012 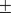 0.004

The impact of adding additional information to the model is listed.

## Discussion

The main goal of this work is to provide a theoretical framework which allows one to determine the quality of the prediction. Conceptually, it is related to the Bayesian approach because it takes into account the impact of additional information as well as the impact of decorrelations on the estimation of future events. As a formal framework we have used a multivariate regression approach.

The prediction of soccer results is a particularly nice case study of this approach due to the availability of well-defined data and due to the popular interest in this matter. Beyond the application of the analytical results it turned out to be essential to search for observables (here: chances for goals) with a high information content.

**Figure 5:**
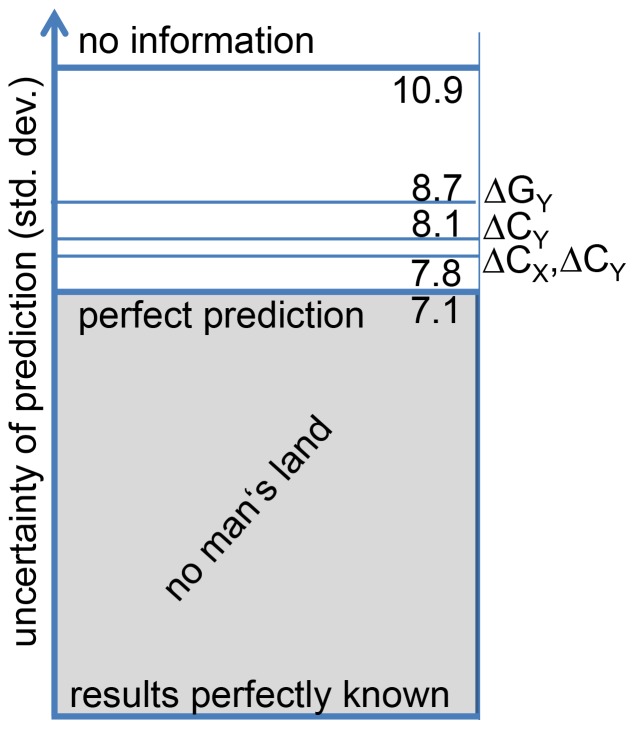
The uncertainty of the prediction of the goal difference of the second half when using the complete information of the first half (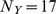). Different choices of variables are shown. Furthermore, the limit of perfect predictability is indicated.

One interesting question arises: is the residual statistical error of 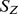 for 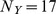 small or large? This question may be discussed from two different perspectives. First, one may want to predict the outcome of the second half of the league. Then the uncertainty is given by 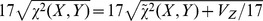. These values are plotted for different prediction scenarios in Fig.5. One can see how the additional information decreases the uncertainty of the prediction. Most importantly, the *no man's land* below an uncertainty of 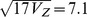 cannot be reached by any type of prediction. The art of approaching this perfect prediction thus resorts to decrease the present value of 7.8 to a value closer to 7.1. Second, one may be interested in the prediction of a single match. This case is somewhat different. Since the team fluctuations are very difficult to predict, the fluctuation amplitude 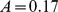 (see above) serves as a scale for estimating the highest possible quality of match prediction. If the uncertainty is much smaller than 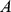 any further improvement would be irrelevant due to the non-predictable fluctuations of the team strength. However, in the present case the statistical error after 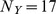 is close to 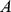 so that a further reduction of 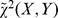 would still be relevant for prediction purposes of individual matches.

Repeating this analysis for the prediction of the points in the second half of the season the statistical uncertainty of the estimation corresponds to approx. 6 points (standard deviation). This corresponds to lose rather than to win two matches or vice versa.

Note that the chances for goals are not a completely objective observable because finally also the subjective judgement of a sports journalist may influence its estimates. In this sense the high information content of chances for goals indicates that the subjective component is quite small and the general definition is very reasonable. Of course, in the future one may look for strictly objective match observables taken by commercial companies to further improve the information content. In any event, the chances for goals are by far more informative than the actual results, as typically taken for prediction purposes.

As demonstrated above, the present results can be directly applied to the prediction of individual soccer matches. The reason is that the team strength, as estimated via the above regression analysis, is the key input for the formalism of single-match prediction, as outlined in Ref. [21], [22].

Of course, it is conceivable that the general ideas can be used for different applications under the condition that very recent (exact but noisy) and more previous information (slightly changed but low noise level) is present. Note that the type of data is identical to panel data, popular in socio-economic studies. Popular cohort studies deal with the time-evolution of the income or the health situation (see, e.g., [25]-[27]). For the testing of stochastic concepts sports data are, of course, particularly suited, because of the easily accessible and reliable data basis.
